# Formation of Oriented Nanowires from Mixed Metal Oxides

**DOI:** 10.3390/ma16196446

**Published:** 2023-09-27

**Authors:** Anna Dikovska, Genoveva Atanasova, Rumen Nikov, Georgi Avdeev, Zara Cherkezova-Zheleva, Daniela Paneva, Nikolay Nedyalkov

**Affiliations:** 1 Institute of Electronics, Bulgarian Academy of Sciences, 72 Tsarigradsko Chaussee, 1784 Sofia, Bulgaria; dikovska@ie.bas.bg (A.D.); rumen_nikov24@abv.bg (R.N.); nned@ie.bas.bg (N.N.); 2 Institute of General and Inorganic Chemistry, Bulgarian Academy of Sciences, Acad. G. Bonchev Str., Bl. 11, 1113 Sofia, Bulgaria; 3 Rostislaw Kaischew Institute of Physical Chemistry, Bulgarian Academy of Sciences, Acad. G. Bonchev Str., Bl.11, 1113 Sofia, Bulgaria; g_avdeev@ipc.bas.bg; 4 Institute of Catalysis, Bulgarian Academy of Sciences, Acad. G. Bonchev Str., Block 11, 1113 Sofia, Bulgaria; zzhel@ic.bas.bg (Z.C.-Z.); daniela@ic.bas.bg (D.P.)

**Keywords:** PLD in open air, *ns* ablation, *ps* ablation, metal oxide composites

## Abstract

In this study, we present a physical method for the fabrication of oriented nanowires composed of mixed metal oxides. Pulsed laser deposition carried out in the air under atmospheric pressure was used for the production of samples. Two sets of experiments were performed by applying nanosecond and picosecond laser ablation, respectively. The depositions were performed using the laser ablation of mixed targets from iron oxide and zinc oxide as the initial materials in different ratios. The experiments were carried out in a magnetic field, which allowed us to control the morphology of nanostructures. The structure, microstructure, morphology, and composition of the structures obtained were studied in relation to the sample composition and laser ablation regime applied. The morphological analysis revealed that the structure of the samples consisted mainly of nanowire-like features reaching tens of micrometers in length. These nanowires were composed of nanoparticles and oriented predominantly in parallel to magnetic field lines. Nanoparticles produced using *ps* ablation were, on average, smaller than those obtained by *ns* ablation of the same target. Using ablation with *ps* laser pulses, we were able to produce new composite materials or materials containing unstable phases.

## 1. Introduction

Iron oxide-containing nanoparticles and nanostructures have long held the attention of researchers and technologists because of their unique physical properties, which provide opportunities for practical applications [1,2]. Magnetite and maghemite are among the most interesting and promising oxides because, at the nanoscale, they exhibit unique magnetic properties, biocompatibility, and biodegradability, and these characteristics make them suitable for applications in medicine [3,4,5,6,7,8]. They can be used as contrast-enhancing elements for biomedical diagnostics [7], in targeted drug delivery [9], for the magnetic isolation and separation of labeled cells [10], etc. Such uses usually require a specific size distribution and the desired shape of the nanoparticles or nanoparticle ensembles since their efficiency strongly depends on their magnetic properties. Producing ordered nanoparticle ensembles, especially magnetic nanowires, has attracted special attention due to their enhanced magnetic properties: shape anisotropy, high magnetic moment, coercivity, and remanent magnetization [11,12,13]. Such nanosized objects should ideally be fabricated through precise size- and shape-controlled synthesis without the use of additional toxic chemicals. However, conventional fabrication methods of iron oxide nanoparticles are chemically based and involve chemical reduction [8], chemical vapor condensation [14], co-precipitation [15], sol-gel [16], etc. Thus, the fabrication of nanoparticles or nanoparticle ensembles in an environmentally friendly manner using a simple and flexible method and conventional low-cost equipment is still a challenge.

A novel and promising physical method for the fabrication of oriented nanostructures of magnetic materials is pulsed laser deposition (PLD) in the air at an atmospheric pressure (PLD in the open air or atmospheric PLD), which is applied in the presence of a magnetic field [17,18,19]. This method allows the production of nanowires composed of nanoparticles arranged along the magnetic force lines. Additionally, the proposed method can be easily modified to fabricate more complicated systems, including composite nanostructures made of magnetic and non-magnetic materials [20,21]. This technology was first developed for *fs*-laser pulses [17]. Nedyalkov and coworkers demonstrated the fabrication of nanoparticle-composed nanowires of magnetic materials such as steel and Ni. This technology was later adapted for *ns* ablation and developed into commercial technology for nanostructure fabrication [18,19]. The main difference between these two techniques is that, in the case of *ns*-laser pulses, the nanoparticles that build the nanowires are formed by the condensation of ablated material [18,19], while ultra-short laser pulses lead to the direct ejection of nanoparticles due to the specific mechanisms of material removal [17].

Industrial applications of *fs* lasers are still limited because of their high price, expensive maintenance, and very specific requirements in the working environment. At the same time, picosecond laser pulse-generating systems with a duration of up to tens of picoseconds are of considerable scientific and commercial interest. In their interaction with the matter, these impulses retain the main characteristics of *fs*-laser pulses: very limited thermal effects, the lack of a broad thermal-affected area around the impact area, and non-thermal mechanisms of phase transformations, such as phase explosion and homogeneous melting. These laser systems are simpler and more stable than *fs* laser systems, leading to their lower cost, which is comparable to that of popular and widely used nanosecond systems. Such lasers could be easily applied in industries. However, due to the specific interaction of radiation with the matter at these pulse durations, the fundamental physical picture of the processes involved is still unclear and needs detailed investigation.

The aim of this work was to fabricate complex ordered nanostructures of iron-containing metal oxides by implementing a physical method, namely PLD, in the open air and the presence of a magnetic field based on ablation using *ns* and *ps* laser pulses. We emphasize the characteristics of this process and the outcomes of *ns* and *ps* ablation employed for the fabrication of nanoparticle-composed nanowires from multicomponent targets. To the best of our knowledge, composite nanowires produced by *ps*-PLD are reported here for the first time. The possibility of obtaining a new composite material or materials containing unstable phases is discussed in view of the different mechanisms of laser–matter interactions using short and ultra-short laser pulses.

## 2. Materials and Methods

### 2.1. Sample Fabrication

Oriented nanowires composed of mixed metal oxides were obtained by PLD in the presence of a magnetic field, as described elsewhere [18,19,21]. The experimental setup was a modification of the classical PLD configuration with a permanent magnet (B = 0.4 T) placed on the back side of the substrate. In such a configuration, the magnetic field lines were parallel to the substrate’s surface [18,19,21]. Two sets of experiments were carried out based on nanosecond (*ns*) and picosecond (*ps*) laser ablation, respectively. The *ns* ablation was performed using 15-*ns* laser pulses delivered by a Nd:YAG (LS-2147, Lotis TII, Minsk, Belarus) laser system at a repetition rate of 10 Hz. The *ps* ablation process was carried out using a picosecond Nd:YAG laser (PS-A1-1064, CNL laser, Changchun, China) with a pulse duration of 10 ps and a repetition rate of 1 kHz. In both cases, the fundamental wavelength of these lasers at 1064 nm was used. The ablation targets were produced by mixing the initial compound of Fe_2_O_3_ (ChemPur, CAS No. 1309-37-1) with ZnO (Merck, CAS No. 1314-13-2) at a different weight percent, as follows: target N0—pure Fe_2_O_3_; target N1—5wt% ZnO in Fe_2_O_3_; target N2—10wt% ZnO in Fe_2_O_3_; target N3—25wt% ZnO in Fe_2_O_3_; target N4—50wt% ZnO in Fe_2_O_3_, and target N5—75wt% ZnO in Fe_2_O_3_. The mixed powders were then homogenized, cold pressed at 5 MPa into tablets and synthesized at 900 °C for 4 h. The laser fluence applied on the targets for *ns* and *ps* ablation was 4 and 0.4 J/cm^2^, respectively. The material ablated from the targets was deposited on quartz or silicon substrates. The distance between the target and substrate was 25 mm. The depositions were carried out in the air under atmospheric pressure.

### 2.2. Sample Characterization

The morphology of the samples was analyzed using scanning electron microscopy (SEM) and a LYRA I XMU system (Tescan, Brno, Czech Republic). Their microstructure was studied via transmission electron microscope (TEM) images taken by a JEOL JEM 2100 system (Akishima-Shi, Tokyo, Japan). Using an Empyrean diffractometer (PANalytical), X-ray diffraction (XRD) was employed to examine the sample crystalline structure and phase composition. The crystalline phases were identified through PAN-ICSD and COD database cards. Transmission Mössbauer spectra were recorded using an electromechanical spectrometer Wissenschaftliche Elektronik (Starnberg, Germany) at a constant acceleration mode and ^57^Co/Rh source (10 mCi). The velocity was calibrated by the α-Fe standard. The parameters of hyperfine interactions in the obtained spectral components (isomer shift ((IS)), quadruple shift ((2ε))/quadruple splitting ((QS)), hyperfine effective field ((B_hf_)), line width ((FWHM)) and partial area ((A)) were determined using the WinNormos program. Computer fitting was based on the least square method. Using an AXIS Supra electron spectrometer (Kratos Analytical Ltd., Manchester, UK) and AlKα radiation with a photon energy of 1486.6 eV, X-ray photoelectron spectroscopy (XPS) was used to determine the surface composition of the samples. The energy calibration was performed by normalizing the C1 line of adsorbed adventitious hydrocarbons to 284.8 eV.

## 3. Results

**Figure 1:**
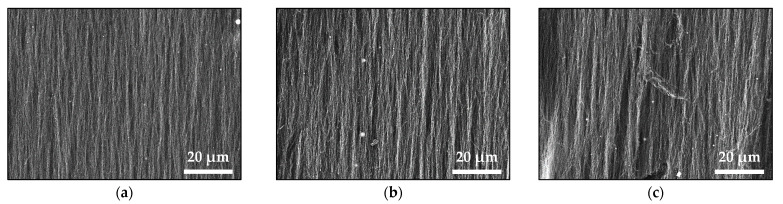

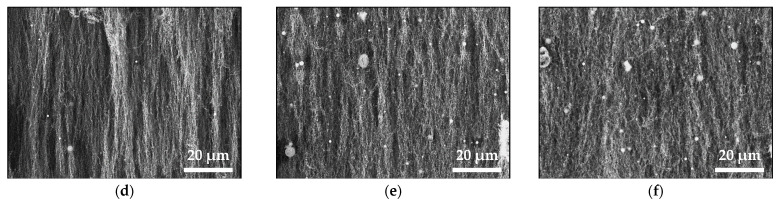
SEM images of the samples produced by *ns* laser ablation of (**a**) Pure Fe_2_O_3_ (N0) and mixed (**b**) N1, (**c**) N2, (**d**) N3, (**e**) N4, and (**f**) N5 targets.

Figure 1 displays the SEM images of samples deposited in a magnetic field via the *ns* laser ablation of the pure Fe_2_O_3_ target (Figure 1a) and mixed Fe_2_O_3_-ZnO targets with different ratios of the initial compounds (Figure 1b–f). The morphological analysis revealed that the structure of these samples consisted mainly of nanowire-like features (hereafter called nanowires) reaching tens of micrometers in length. These nanowires tend to be oriented parallel to the external magnetic field lines, with this orientation diminishing with an increase in the ZnO content in the target. It should be noted that the oriented nanowires formed bundles, as seen in Figure 1a–d, and these bundles disappeared with the loss of nanowire orientation. In addition, some randomly distributed droplets, typical for the *ns*-PLD technology, were also observed; their numbers increased along with the ZnO content in the target.

**Figure 2:**
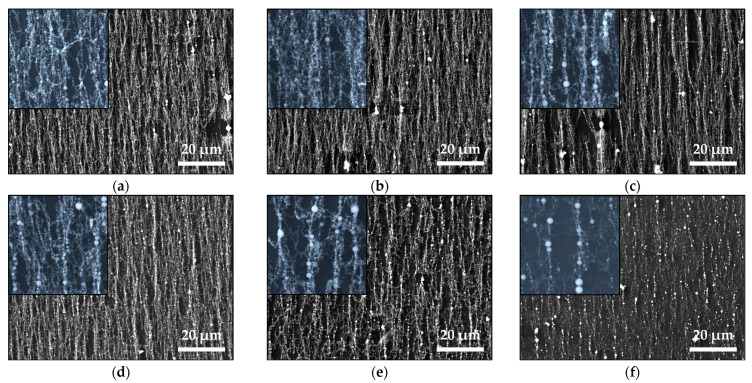
SEM images of the samples produced using *ps* laser ablation of (**a**) pure Fe_2_O_3_ (N0) and mixed (**b**) N1, (**c**) N2, (**d**) N3, (**e**) N4, and (**f**) N5 targets. Insets are SEM images at a higher magnification with the size 10 × 10 μm.

Figure 2 shows the SEM images of samples deposited from Fe_2_O_3_ and mixed with Fe_2_O_3_-ZnO targets using *ps* laser ablation. The presence of vertically oriented features parallel to magnetic field lines is evident in all the images. The nanowire length is in the range of microns. The insets show the detailed structure of the nanowires. They are composed of mostly spherical nanoparticles of different sizes. Particles with diameters in the range of 150–450 nm and even larger can be clearly distinguished; their number increased with the increase in ZnO content in the targets (Figure 2d–f). It is also evident that the total amount of material deposited on the substrate decreased with the increase in ZnO in the targets. However, bundles of oriented nanowires can still be observed even though the target with the highest ZnO content ablated (Figure 2f).

**Figure 3:**
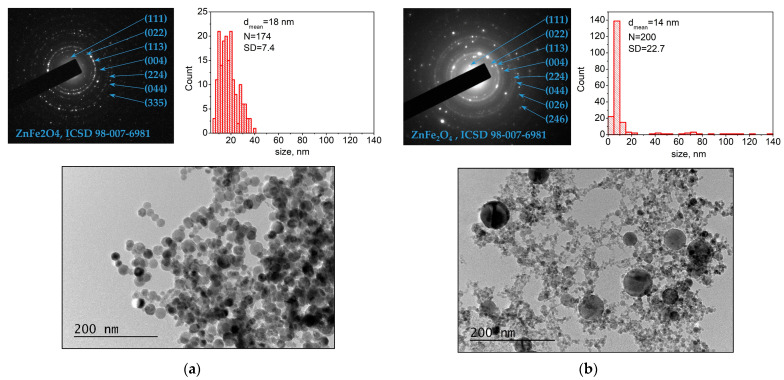
TEM image with corresponding SAED pattern and size distribution of the sample deposited by (**a**) *ns* ablation and (**b**) *ps* ablation from target N4.

A TEM image of a sample deposited using *ns* ablation from target N4 is shown in Figure 3a. The sample microstructure can be seen as polygon-shaped nanoparticles with different sizes. The size distribution is also presented in Figure 3a; nanoparticle size was in the range of 5–40 nm with a mean diameter of 18 nm. SAED image demonstrated that the nanoparticles were crystalline. The TEM images of samples deposited from other targets display microstructures resembling those in Figure 3a, i.e., with a similar shape and size distribution of nanoparticles. Figure 3b shows a TEM image of a sample deposited using *ps* ablation from target N4. The microstructure of the sample consists of spherical and polygon-shaped nanoparticles. Particles with sizes from approximately 40 nm to 120 nm, as well as smaller ones with sizes in the range of 2–20 nm, can be clearly distinguished (see the size distribution in Figure 3b). The nanoparticles are crystalline with a mean diameter estimated at 14 nm (size distribution in Figure 3b). The microstructure of the samples deposited by the *ps* ablation of the other targets did not differ significantly from that of the sample deposited from target N4.

**Figure 4:**
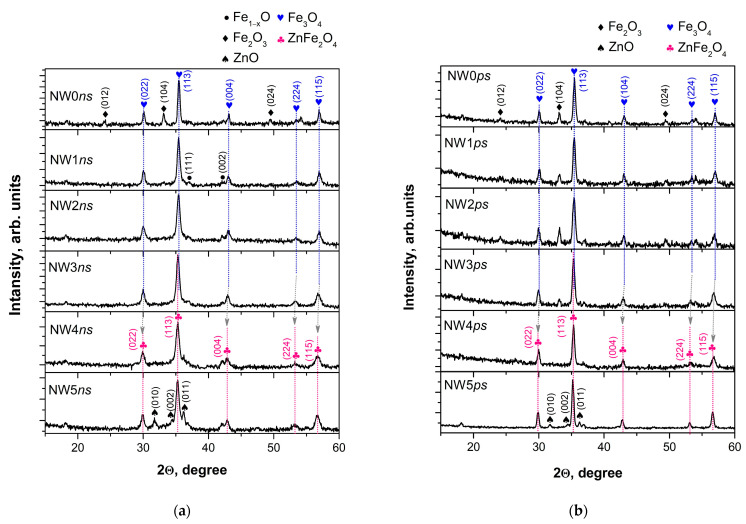
XRD patterns of samples produced by the (**a**) *ns* and (**b**) *ps* laser ablation of different targets. The samples (nanowires) deposited from target N0 are named NW0; from N1 as NW1; from N2 as NW2; from N3 as NW3; from N4 as NW4, and from N5 as NW5.

The XRD patterns of the samples (nanowires) deposited using the *ns* laser ablation of Fe_2_O_3_ and mixed targets are presented in Figure 4a. The phase composition of the sample deposited from Fe_2_O_3_ is a combination of iron oxides, i.e., magnetite (Fe_3_O_4_, ICSD 98-015-8741), hematite (α-Fe_2_O_3_, ICSD 98-005-6372) and wüstite (Fe_1−x_O, ICSD 98-002-7237), as previously reported [15,16]. The phase composition of the sample deposited from target N1 (with the smallest ZnO content in the initial material) did not differ from the composition of the sample deposited from the pure Fe_2_O_3_ target; the presence of ZnO or other Zn-containing compounds in the sample was not identified. Increasing the Zn content into the target led to the deposition of samples with a slightly higher magnetic phase content, i.e., magnetite, and a lower content of other, non-magnetic, iron oxides (sample deposited from target N2). This tendency continued with the further Zn content increase in the target (deposition from target N3). The presence of a Zn-containing phase was clearly expressed in the sample deposited from target N4. The phase composition of the sample was identified as zinc ferrite (ZnFe_2_O_4_, ICSD 98-007-6981). Lastly, ablating the target with the highest Zn content led to the deposition of composite samples consisting of ZnFe_2_O_4_ and ZnO. The lattice parameters of the main phase of samples deposited from mixed targets are presented in Table 1.

The reference values of magnetite and zinc ferrite are also present. As seen, increasing the Zn content in the targets also increased the lattice parameter of the main phase of the sample. Figure 4b reports the XRD patterns of samples deposited by the *ps* laser ablation of Fe_2_O_3_ and mixed targets. The *ps* laser ablation of the Fe_2_O_3_ target led to the deposition of samples consisting of magnetite and hematite phases of iron oxides, with the predominant phase being magnetite. The content of the magnetic phase in the samples increased with the increasing ZnO in the targets (deposition from targets N1, N2, and N3) at the expense of the non-magnetic phase of hematite. Meanwhile, the lattice parameter of the samples’ main phase increased, as seen in Table 1. The Zn-containing phase clearly appeared in the sample deposited from target N4; the phase composition of the sample was identified as zinc ferrite. The further increase in the Zn content into the target (depositions from target N5) led to the deposition of composite samples of ZnFe_2_O_4_ and ZnO.

Table 2 summarizes the data calculated after analyzing the decomposition of the Mössbauer spectra of samples deposited using *ns* ablation. The recorded spectra at room temperature of the investigated materials revealed the presence of sextet spectra components superimposed with doublet ones. The appearance of several magnetically split components (sextets) can be resolved in the spectra of samples with different iron/Zn concentrations and samples deposited from the targets N0, N1, and N2. The major contribution of the sextet Mössbauer components is characteristic of the presence of Fe_3_O_4_ predominantly and α-Fe_2_O_3_ in studied materials (Table 2) [22,23]. The presence of doublet patterns could be assigned to the contribution of Fe-bearing phases in non-stoichiometric Fe_1-x_O and ZnFe_2_O_4_ [24,25].

Table 3 shows the data estimated after the decomposition of Mössbauer spectra in the samples obtained using *ps* ablation. The Mössbauer patterns reveal the different superpositions of sextets and doublet spectra determined by the target used for ablation. The major contribution of sextet components could be assigned to the presence of Fe_3_O_4_ predominantly and α-Fe_2_O_3_ in samples deposited from targets N0–N3. The presence of doublet patterns is characteristic of the contribution of ZnFe_2_O_4_, as this phase presents in the spectra of all samples deposited from the target containing ZnO as an initial material. Also, the presence of γ-Fe_2_O_3_ is clearly recognized in the spectra of samples deposited from targets N4 and N5.

**Figure 5:**
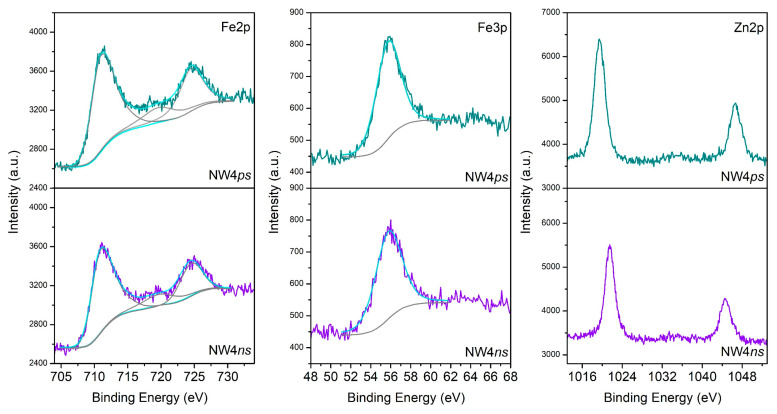
XPS spectra of samples deposited via ns and ps ablation from target N4.

XPS analysis of the surface of samples deposited from target N4 via ns and ps ablation is presented in Figure 5. As seen, the samples’ binding energies for Fe2p_3/2_ and Fe2p_1/2_ were 711 eV and 724.5 eV, respectively. The Fe2p_3/2_ peak had an associated satellite peak situated at 719.6 eV. We can conclude that Fe atoms were in the Fe^3+^ oxidation state due to the estimated spin-orbit splitting of 13.6 eV and the binding energy positions, as well as the presence of a satellite peak at 719.6 eV [26,27]. No peak typical of Fe^2+^ (~53.7 eV) was detected [27]. The Zn 2p_3/2_ and Zn 2p_1/2_ peaks were seen at 1021.5 eV and 1044.6 eV, respectively, with a spin-orbit splitting of 23.1 eV. The width of the Zn 2p peaks, the binding energy positions, and the spin-orbit splitting all pointed to Zn atoms being in the Zn^2+^ oxidation state [26]. The asymmetric O 1s peak (not shown) implied the presence of different oxygen-containing species. This peak could be deconvoluted into two components: at nearly 529.9 eV, attributed to lattice O^2-^ ions in the metal oxides, and 532.7 eV, attributed to the presence of adsorbed hydroxyl, carbonate, or O_2_ species [27,28,29]. No substantial difference is seen between the spectra of the samples NW4ns and NW4ps presented in Figure 5.

Table 4 summarizes the Fe/Zn ratio on the sample surface against the respective mixed targets used for depositions. It can be concluded that increasing the Zn content in the targets reduced the Fe/Zn ratio on the sample surface.

## 4. Discussion

The phase composition of mixed targets is a combination of hematite and zinc ferrite, as shown in Figure S1 and Table S1, depending on the initial content of ZnO. The predominant hematite phase decreases and disappears in favor of ZnFe_2_O_4_ with the increase in ZnO’s weight percentage in the targets. Target N4, obtained from the equal weight percentage of Fe_2_O_3_ and ZnO, is identified as a zinc ferrite phase. It is not surprising that the structure of the sample deposited from target N4, regardless of the ablation regime, consisted of ZnFe_2_O_4_ because the main advantage of the pulsed laser deposition process is the potential for the stoichiometric transfer of material from the target to the substrate. The predominant phase of samples deposited from targets with a lower ZnO content is magnetite, and no Zn-containing compounds could be identified in the XRD patterns (Figure 4). However, the presence of Zn and/or Zn-containing compounds was confirmed by XPS as well as by Mössbauer analyses. The results from XPS analyses show that the decrease in the Fe/Zn ratio on the sample surface correlates with the rise in the ZnO content in the targets (Table 4). It is worth noting that the sample fabrication technology applied here was a kind of sputtering process, i.e., a physical vapor deposition process, which implies that the composition analyzed on the sample surface is highly unlikely to change in-depth if the target used is homogeneous. In this light, it could be concluded that with the increase in the zinc content in the target, Zn^2+^ tends to substitute Fe^2+^ in Fe_3_O_4_, producing zinc ferrite. This tendency is already evident in samples from targets with a low ZnO concentration (N1–N3) and persists to the highest concentrations since ZnFe_2_O_4_ is identified as the main phase in the sample deposited from target N5: a target with a higher initial content of ZnO than Fe_2_O_3_. The change in the lattice parameter of magnetite with the increase in the target ZnO content (Table 1) supports this interpretation. Considering the XRD patterns shown in Figure 4, the main difference in the crystal structure of the samples deposited via *ns* and *ps* ablation is the presence/absence of Fe_1−x_O. The non-stoichiometric wüstite phase is present in the samples prepared using *ns* ablation but cannot be found in the samples deposited via *ps* ablation from the same targets. We attribute this result to the mechanism of the laser–matter interaction during the so-called short (*ns*) and ultra-short (<10 ps) laser pulses and the further evolution of ablated material in the air at atmospheric pressure [30]. In the classical *ns* ablation process, a laser pulse rapidly heats the target. Thus, during *ns* ablation, a significant part of α-Fe_2_O_3_ is reduced to FeO, which further participates in the formation of the Fe_3_O_4_ phase (FeO·Fe_2_O_3_) [18]. The presence of Fe-containing material allows the potential use of Mössbauer spectroscopy for the precise identification of the sample phase composition. The Mössbauer results for the samples deposited via *ns* ablation are in good agreement with XRD analysis (Table 2 and Figure 4a). The exceptions observed can be related to the distinctive features of the Mössbauer method to register amorphous phases together with crystalline ones, revealing only iron-containing compounds. This latter point means that only the ZnFe_2_O_4_ phase could be registered in the sample deposited from target N5 via *ns* ablation. The possibility of the presence of an amorphous phase is ruled out by the microstructural analysis (Figure 3a), which demonstrates the crystalline nature of the sample. Furthermore, no amorphous halo was observed in any of the XRD patterns shown in Figure 4a. Hence, on the one hand, we refer to the negligible differences in sample composition as obtained by XRD and Mössbauer spectroscopy to the error in quantifying the phase composition and the fabrication method repeatability on the other. In the case of *ps* ablation, the microstructure of the sample is polycrystalline, as revealed from the SAED pattern (Figure 3b). However, the TEM image and the corresponding size distribution of the sample show a significant presence of nanoparticles with very small sizes, in the range of 1–4 nm. The XRD method does not recognize such small particles as crystalline, and they are registered as an amorphous halo in the XRD pattern (Figure 4b). In this regard, we assumed that the differences between XRD and Mossbauer results concerning the sample phase composition are due to the presence of small-sized nanoparticles obtained via *ps* ablation. It should be noted that the precise identification of sample phase composition using Mössbauer spectroscopy shows the presence of maghemite (γ-Fe_2_O_3_). The maghemite phase is a polymorph form of hematite with a spinel structure-like magnetite [14]. Since this phase is unstable, it transforms into hematite at temperatures between 250 and 750 °C. In this way, using ablation via *ps* laser pulses due to the shorter laser–matter interaction time compared to the *ns* ablation process, we were able to produce an iron oxide phase such as the maghemite, which is difficult to obtain via the sputtering process. Further, it should be noted that the relative weights of the ZnFe_2_O_4_ phase in the samples deposited using the *ps* laser seem not to correspond to the increase in Zn-containing components in the targets (Table 3 and Table S1). This indicates that a part of ZnFe_2_O_4_ is decomposed to magnetite or maghemite. It should be emphasized that zinc ferrite is a mixed metal oxide, which, in its bulk form, typically has a paramagnetic behavior. However, prepared at the nanometer scale, ZnFe_2_O_4_ exhibits ferromagnetic/ferrimagnetic properties [31]. It can be summarized that the material deposited by the open-air ablation of mixed targets, regardless of the initial content of Fe_2_O_3_ and ZnO powders in the target, is a composite with a predominant magnetic phase—magnetite, maghemite, or zinc ferrite.

After the initial ejection of material from the target via *ns* or *ps* ablation and plasma plume formation, the ablated material further evolves in the air at atmospheric pressure. In the case of *ns* ablation, the process taking place in the open air results in the formation of nanoparticles/nanoparticle aggregates in the plasma plume due to the ablated material condensation [18,19]. In the case of *ps* ablation, ultrashort laser pulses result in the direct formation of nanoparticles due to their fragmentation and/or phase explosion, leading to material removal [17]. As a consequence, regardless of the ablation regime applied, the “building blocks” of the samples deposited on the substrate are nanoparticles and/or nanoaggregates with different sizes and shapes, as is evident from the TEM images presented in Figure 3.

Since the atmospheric pressure (as a high surrounding pressure) confines the plume and limits its size to a few millimeters, the ablated material can undergo phase-composition transformations and nanoparticle agglomeration since the nanoparticles formed rapidly decelerate. The small-sized plasma plume means that the evolution of ablated material occurs close to the target; consequently, the ablation process in the open air is an inefficient process. As was previously reported, no material deposited on the substrate is observed or is barely observed for target–substrate distances larger than 5 mm [18]. However, the amount of deposited material significantly increases when a magnetic component is present in the plasma plume and a suitable external magnetic field is applied [18,19]. The external magnet placed at the backside of the substrate attracts the magnetic nanoparticles and nanoaggregates, arranges them in parallel to the magnetic field lines, and collects them on the substrate [18,19,20,21]. However, for the cases reported here, XRD and Mössbauer results confirm the presence of non-magnetic particles in the structure of the samples (Figure 4, Table 2 and Table 3). Presumably, due to the strong confinement of the plasma plume, the magnetic nanoparticles carry away or coalesce with non-magnetic ones and transport them to the substrate [20,21]. The presence of a magnetic phase is clearly confirmed by the SEM images shown in Figure 1. Micron-sized nanowires were present in all the samples produced via *ns* and *ps* laser ablation. A detailed look at the morphology of the samples deposited from the same target using *ns* and *ps* laser ablation shows differences, which we can attribute to their different microstructure (Figure 3). The nanowires produced via *ns* ablation are composed of nanoparticles with similar sizes (Figure 3a), and separate nanoparticles are not clearly distinguished in the nanowires (Figure 1). By contrast, in *ps* ablation, it is clearly seen that distinct nanoparticles are arranged in nanowires (insets of Figure 2). We can visually recognize nanoparticles of different sizes arranged into nanowires, reflecting the broad range of nanoparticle size distribution (SD = 22.7) shown in Figure 3b. The presence of small nanoparticles also indicates that the magnetic moment induced by the external magnetic field is small since it is proportional to the size (or, more precisely, the volume) of the nanoparticles [17]. Subsequently, the smaller nanoparticles produced via *ps* ablation are weakly or not at all attracted by the magnetic field compared to the nanoparticles deposited via *ns* ablation from the same target. This explains the smaller amount of material deposited by *ps* ablation (Figure 1 and Figure 2).

Although the phase composition of the samples was identified as a combination of different iron-containing oxides, the surface analysis showed that Fe atoms were in the Fe^3+^ oxidation state. We assumed that due to passivation on the surface in open air, the iron oxide nanoparticles were oxidized to Fe_2_O_3_ [32].

To summarize, oriented nanowires composed of magnetic metal oxides can be easily prepared by pulsed laser deposition in the presence of a magnetic field [33]. The various process parameters, such as laser fluence, target–substrate distance, ambient atmosphere and pressure, and magnetic field, all affect the structure and morphology of the nanostructures produced, as has been previously studied and reported [18,19]. Similar effects of process parameters on the samples’ structural and morphological properties also take place when a multicomponent target (targets in our work) is used for ablation. The use of multicomponent targets composed of different and/or mixed oxides additionally widens the possibility of fabricating new composite materials or materials composed of unstable phases. However, it should be noted that when ultrashort lasers are used for ablation, a critical parameter for the fabrication of such nanowires is the size of the laser spot. When the area of the laser spot is a few hundred μm^2^ (typical for ultrashort lasers) [34], the quantity of ablated material is too small for efficient interaction in the plasma plume, and consequently, only large particles are deposited on the substrate. Such material deficiency could be easily overcome by increasing the pulse repetition rate, as demonstrated in our study.

## 5. Conclusions

Micron-sized composite nanowires were produced via *ns* and *ps* laser ablation in the air at atmospheric pressure in the presence of an external magnetic field. Nanowires fabricated using *ns* ablation were composed of nanoparticles with similar sizes, and separate nanoparticles were not clearly distinguished, while nanoparticles of different sizes arranged into nanowires could be visibly recognized when *ps* ablation was applied. We can explain the different sample morphology with the different microstructures of the samples deposited using *ns* and *ps* laser ablation. The laser ablation of multicomponent targets composed of iron oxide and zinc oxide in different ratios as initial materials led to the fabrication of composite materials consisting of Fe-oxides and Zn-ferrite. Based on the Mössbauer results, it can be summarized that the Fe_1-x_O phase is identified in the phase composition of the samples deposited via *ns* ablation, while *ps* ablation leads to the presence of γ-Fe_2_O_3_ in the sample composition. Using ablation with *ps* laser pulses, due to the shorter laser–matter interaction time compared to the *ns* ablation process, we were able to produce the maghemite iron oxide phase, which is hard to obtain through a sputtering process. Furthermore, using the *ns* ablation of a multicomponent target, the stoichiometric transfer of material from the target to the substrate can be implemented. Using the *ps* ablation of the same target, this stoichiometric transfer of material from the target to the substrate was no longer possible since part of ZnFe_2_O_4_ was decomposed to magnetite or maghemite. Fabricating chemically clean magnetic nanowires with desirable composition using a simple and flexible method and conventional low-cost equipment makes their application possible in the field of biomedicine.

## Figures and Tables

**Table 1:** Lattice parameter of the main phase of samples deposited from different targets by *ns* and *ps* laser ablation.

Sample Deposited from Target		Fe_3_O_4_, ICSD 98-015-8741	N0	N1	N2	N3	N4	N5	ZnFe_2_O_4_, ICSD 98-007-6981
Lattice parameter, Å	*ns* ablation	8.3860	8.396 (3)	8.407 (4)	8.415 (4)	8.414 (8)	8.423 (8)	8.432 (8)	8.4220
	*ps* ablation		8.388 (2)	8.389 (2)	8.394 (2)	8.417 (2)	8.416 (3)	8.437 (2)	

**Table 2:** Calculated parameters after fitting the Mössbauer spectra of samples deposited by *ns* ablation from different targets. The parameters are as follows: isomer shift (IS), quadruple shift (2ε)/quadruple splitting (QS), hyperfine effective field (B_hf_), line width (FWHM) and partial area (A).

Sample	Components	IS,	2ε/QS,	B_hf_,	FWHM,	A,
		mm/s	mm/s	T	mm/s	%
NW0*ns*	Sx1-α-Fe_2_O_3_	0.36	−0.17	51.2	0.25	7
	Sx2-Fe_3−x_O_4_	0.27	0.02	48.7	0.39	34
	Sx3-Fe_3−x_O_4_	0.64	0.03	44.9	0.58	43
	Db1-Fe_1−x_O	0.98	1.02	-	0.50	16
NW1*ns*	Sx1-α-Fe_2_O_3_	0.36	−0.22	51.6	0.30	8
	Sx2-Fe_3−x_O_4_	0.29	0.01	48.9	0.35	26
	Sx3-Fe_3−x_O_4_	0.61	0.03	45.1	0.67	46
	Db1-Fe_1−x_O	0.90	0.78	-	0.68	14
	Db2-ZnFe_2_O_4_	0.35	0.42	-	0.50	6
NW2*ns*	Sx1-Fe_3−x_O_4_	0.28	0.01	47.9	0.30	15
	Sx2-Fe_3−x_O_4_	0.66	0.01	45.8	0.52	30
	Db1-Fe_1−x_O	1.10	0.41	-	0.40	19
	Db2-ZnFe_2_O_4_	0.32	0.38	-	0.44	36
NW3*ns*	Db1-Fe_1−x_O	1.11	0.40	-	0.30	18
	Db2-ZnFe_2_O_4_	0.32	0.41	-	0.34	82
NW4*ns*	Db1-Fe_1−x_O	1.12	0.09	-	0.50	12
	Db2-ZnFe_2_O_4_	0.32	0.48	-	0.40	88
NW5*ns*	Db-ZnFe_2_O_4_	0.32	0.45	-	0.46	100

**Table 3:** Calculated parameters after fitting the Mössbauer spectra of samples deposited via *ps* ablation from different targets. The parameters are as follows: isomer shift (IS), quadruple shift (2ε)/quadruple splitting (QS), hyperfine effective field (B_hf_), line width (FWHM) and partial area (A).

Sample	Components	IS,	2ε/QS,	B_hf_,	FWHM,	A,
		mm/s	mm/s	T	mm/s	%
NW0*ps*	Sx1-α-Fe_2_O_3_	0.36	−0.21	51.5	0.26	26
	Sx2-Fe_3−x_O_4_	0.28	0.01	48.9	0.34	31
	Sx3-Fe_3−x_O_4_	0.64	0.00	45.7	0.45	36
	Db-Fe^3+^	0.32	0.49	-	0.35	7
NW1*ps*	Sx1-α-Fe_2_O_3_	0.36	−0.20	51.5	0.29	45
	Sx2-Fe_3−x_O_4_	0.28	0.02	48.9	0.32	17
	Sx3-Fe_3−x_O_4_	0.61	0.02	45.1	0.51	29
	Db-ZnFe_2_O_4_	0.33	0.43	-	0.35	9
NW2*ps*	Sx1-α-Fe_2_O_3_	0.34	−0.19	52.1	0.26	15
	Sx2-Fe_3−x_O_4_	0.27	0.01	49.3	0.35	28
	Sx3-Fe_3−x_O_4_	0.57	0.02	45.8	0.66	48
	Db-ZnFe_2_O_4_	0.33	0.42	-	0.45	9
NW3*ps*	Sx1-α-Fe_2_O_3_	0.37	−0.19	51.8	0.45	5
	Sx2-Fe_3−x_O_4_	0.29	0.01	49.4	0.50	27
	Sx3-Fe_3−x_O_4_	0.62	0.02	45.8	0.71	50
	Db-ZnFe_2_O_4_	0.34	0.43	-	0.40	18
NW4*ps*	Sx-γ-Fe_2_O_3_	0.34	0.02	47.9	0.40	7
	Db-ZnFe_2_O_4_	0.34	0.36	-	0.32	93
NW5*ps*	Sx-γ-Fe_2_O_3_	0.34	0.03	47.3	0.30	23
	Db-ZnFe_2_O_4_	0.33	0.44	-	0.67	77

**Table 4:** Fe/Zn ratio on the surface of samples deposited using *ns* and *ps* ablation from different targets.

Sample Deposited from Target		N1	N2	N3	N4	N5
Sample Fe/Zn ratio	*ns* ablation	10.3	6.7	2.3	1.4	1.1
	*ps* ablation	13.4	6.3	3.1	1.9	1.3

## Data Availability

Not available.

## Supplementary Materials

The following supporting information can be downloaded at: https://www.mdpi.com/article/10.3390/ma16196446/s1, Figure S1: XRD patterns of the targets used for ablation: (a) N0 (Fe_2_O_3_) and mixed: (b) N1, (c) N2, (d) N3, (e) N4, (f) N5 target. Table S1: Phase composition of the targets used for ablation.
