# Supplementary material for: Formation of Oriented Nanowires from Mixed Metal Oxides
Source: Materials (Basel). 2023 Sep 27;16(19):6446. doi: 10.3390/ma16196446 (PMC10573213; doi:10.3390/ma16196446)
Supplement: Supplementary file 1 [file materials-16-06446-s001.zip › materials-2602664-supplementary.pdf]

Supplementary Materials:

Article

Formation of Oriented Nanowires from Mixed Metal Oxides

Anna Dikovska [1], Genoveva Atanasova [*2], Rumen Nikov [1], Georgi Avdeev [3], Zara Cherkezova-Zheleva [4], Daniela Paneva [4] and Nikolay Nedyalkov [1]

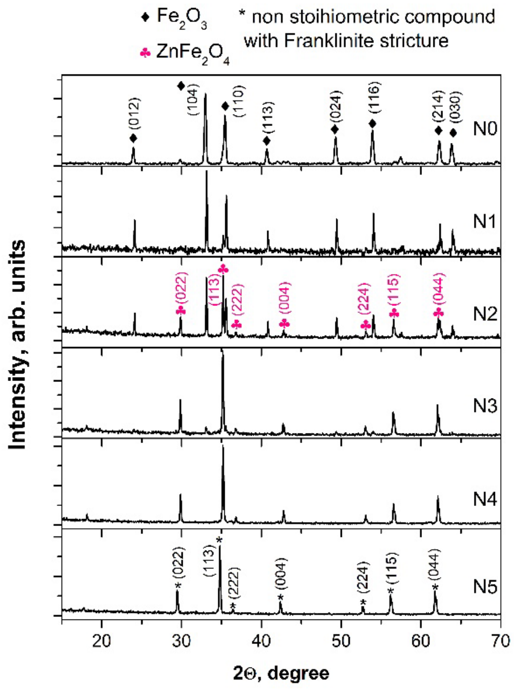

Figure S1. XRD patterns of the targets used for ablation: **(a)** N0 (Fe_2O_3) and mixed: **(b)** N1, **(c)** N2, **(d)** N3, **(e)** N4, **(f)** N5 target.

Table S1. Phase composition of the targets used for ablation.

Phase composition \ Target	N0	N1	N2	N3	N4	N5
Fe_2O_3, %	100	86.7	59.9	15.2	-	-
$ZnFe_2O_4$, %	-	13.3	40.1	84.8	100	-
non-stoichiometric compound with Franklinite stricture, %	-	-			-	100
